# Palm Oil on the Edge

**DOI:** 10.3390/nu11092008

**Published:** 2019-08-26

**Authors:** Eva Gesteiro, Luis Guijarro, Francisco J. Sánchez-Muniz, María del Carmen Vidal-Carou, Ana Troncoso, Lluis Venanci, Vicente Jimeno, Joan Quilez, Arturo Anadón, Marcela González-Gross

**Affiliations:** 1 ImFine Research Group, Departamento de Salud y Rendimiento Humano, Facultad de Ciencias de la Actividad Física y del Deporte-INEF, Universidad Politécnica de Madrid, 28040 Madrid, Spain; 2 Departamento de Periodismo II, Facultad de Ciencias de la Información, Universidad Complutense de Madrid, 28040 Madrid, Spain; 3 Departamento de Nutrición y Ciencia de los Alimentos, Facultad de Farmacia, Universidad Complutense de Madrid, 28040 Madrid, Spain; 4 Departamento de Nutrición y Bromatología, Campus de l’Alimentació de Torribera, Facultat de Farmàcia i Ciències de l’Alimentació, Universitat de Barcelona, 08921 Santa Coloma de Gramenet, Barcelona, Spain; 5 Departamento de Nutrición y Bromatología, Toxicología y Medicina Legal, Universidad de Sevilla, 41012 Sevilla, Spain; 6 Agronomic Engineer, MBA, Independent consultant. El Prat de Llobregat 08820 Barcelona, Spain; 7 Departamento de Producción Agraria, ETSI Agronómica, de Alimentación y Biosistemas, Universidad Politécnica de Madrid, 28040 Madrid, Spain; 8 Europastry S.A. 08210 Barberà del Vallès, Barcelona, Spain; 9 Departamento de Farmacología y Toxicología, Facultad de Veterinaria, Universidad Complutense de Madrid, 28040 Madrid, Spain; 10 Head of ImFine Research Group, Departamento de Salud y Rendimiento Humano, Facultad de Ciencias de la Actividad Física y del Deporte-INEF, Universidad Politécnica de Madrid, 28040 Madrid, Spain

**Keywords:** palm oil, sustainability, 3MCPD, palmitic acid, cardiovascular disease, food industry, communication

## Abstract

Internationally recognized Spanish experts in the food industry, nutrition, toxicology, sustainability, and veterinary science met in Madrid on July 2018 to develop a consensus about palm oil (PO) as a food ingredient. Their aim was to provide a useful, evidence-based point of reference about PO. Scientific evidence about the role of PO in food safety, nutrition and sustainability was analyzed. Main conclusions were: (1) RSPO foundation responded to the environmental impact of palm crops. The Amsterdam Declaration pursues the use of 100% sustainable PO in Europe by 2020. Awareness about choosing sustainable products will help to maintain local economies and environments in the producing countries; (2) evidence shows that a moderate intake of PO within a healthy diet presents no risks for health. No evidence justifies any change fat intake recommendations; (3) food industry is interested in assuring safe, sustainable and high-quality products. The use of certified sustainable PO is increasing; and (4) there is no evidence associating PO consumption and higher cancer risk, incidence or mortality in humans. Tolerable daily intake (TDI) for toxic contaminants (2-and 3-monochloropropanediols (MCPDs), glycidyl esters (GEs)) have been established by JECFA and EFSA. Consequently, the European Commission has modified the Contaminants Regulation for GEs and it is still working on 3-MCPDs’.

## 1. Introduction: Justification and Need of the Consensus

Oil palm has been cultivated for more than 5000 years. Palm oil is the most commonly used vegetable oil in the world today [1]. Palm fruit oil, generally known as palm oil, is produced from the pulp of the fruit of the oil palm (*Elaeis guineensis*) [2]. This tropical fruit is reddish due to its high β-carotene content. The fruit is between 3 and 5 cm long and has a single seed or walnut, which is used to produce palm nut oil, also called palm kernel oil [3]. Each palm fruit contains in total around 30%–35% of oil. The legal denomination of this product in Spain according to Royal Decree 1011/1981 of 10 April approving the Technical sanitary regulation for the elaboration, circulation and trade of edible fats (animal, vegetable and anhydrous), margarines, minarines and fatty preparations [4] is “palm butter”; however, we use the terms “palm oil” or “palm fat” throughout this document, as the names used most often in Spain and at international level.

Palm oil and palm kernel oil have the same botanical origin, but differ significantly in their fatty acid (FA) composition. According to the United States Department of Agriculture (USDA), in 2017/18 growth of world palm oil production closed at 7% above the previous year, rising from 65.25 million tons (Mt) in the year 2016/17 to about 70 Mt in 2017/18, the main producers being Malaysia and Indonesia. Of total world production, 5% is used for biofuels, 24% for cosmetics, and 71% by the food industry. For its part, in the European Union (EU), 87% of imports went to the manufacture of biodiesel. In the year 2000, the EU consumed a bare 271,000 metric tons of palm oil, rising to 3.9 Mt in 2017, with an average annual increase of 7%. The last year saw a downward trend in imports and consumption in the EU, especially palm oil for food and household use, while palm oil for industrial use remained stable [1]. In the food industry, palm oil is used primarily to replace animal fats and hydrogenated fats. Be it remembered that as a result of the research conducted by Keys in the Seven Countries Study [5], and others [6], the intake of saturated animal fats was associated with increased cardiovascular risk (CVR). In view of these findings, during the 1990s animal fats (lard, butter) began to be replaced by others. In these years, the hydrogenation of oils for margarine was seen by the industry as a good solution for obtaining solid or semi-solid fats with a variety of possibilities for melting/crystallization dynamics and for melting temperatures. Between 2000 and 2010, there was growing awareness of the negative effects of trans-FAs on cardiovascular health, also associated with increased risk of atherosclerosis due to the increase in the fraction of cholesterol transported by low-density lipoproteins (LDLc) and reduction of the cholesterol transported by high-density lipoproteins (HDLc). Although there are metabolism-derived trans-FAs in ruminant meat and dairy products, the negative aspects noted earlier have been particularly associated with industrial trans-FAs sources obtained by partial hydrogenation of vegetable oils. The White Paper on nutrition in Spain considers that the highest tolerable intake (upper limit UL) for trans-FAs must be less than 1% of the total energy consumed [7]. In the same vein, the Spanish Federation of Nutrition, Food, and Dietetics Societies (FESNAD) indicates that the intake of trans-FAs must be as low as possible and not exceed 1% of the total energy [8]. The scientific report of the European food safety authority (EFSA) on trans-FAs recommends the lowest possible content in food [9]. The World Health Organization (WHO) recommends that total intake of trans-FAs should not exceed 1% of the total energy of the diet, which is equivalent to less than 2.2 g/day for a 2000 kcal diet. Currently, the “REPLACE program” is being introduced to eliminate trans-FAs in foods on a global basis [10]. Margarines, which emerged as a substitute for animal fats, especially butter and are produced through partial hydrogenation of vegetable oils, contain trans-FAs. So, for the industry, once hydrogenation fats could no longer be used to harden fats, the alternative was palm fat, creating a circular dynamic between increased demand, greater production, increased supply, lower prices, and volatility in retail prices. What *a priori* seemed a reasonable replacement of one fat solution by another is now a subject of wide-ranging debate, even transcending scientific and technical boundaries and reaching consumers through social networks.

The consumption of palm oil is often questioned for various reasons: on the one hand, its effect on cardiovascular health because of its composition; on the other, food security due to the possible accrual of pollutants during refining; and finally, there is the debate on the sustainability of its cultivation in the countries of origin. The food and nutrition sectors are increasingly adopting a more holistic view, which encompasses not only health, but also environmental protection and traceability. The EU promotes these with campaigns such as “From Farm to Fork” (tracing food from production to the table) [11], and the plan of action for the circular economy “Closing the loop” [12]. The circular economy is an economic concept that interconnects with sustainability; its purpose is to ensure that the value of products, materials and resources is maintained in the market for as long as possible, as well as minimizing the generation of waste for disposal. As indicated by Marangoni et al., the absence of any pronouncement from the scientific community regarding the effects of palm oil on consumer health has meant that the message reaches the general public mainly through the mass media and is often skewed and incomplete [3]. In light of the concerns observed among the Spanish and European consumers, it seemed timely to conduct a multidisciplinary forum with ten scientific experts in areas related to the topic (food, human and animal nutrition, dietetics, physical activity, toxicology, sustainability, and public health) with a view to analyzing the existing knowledge, defining research needs and agreeing on what data the message should convey to the consumer. The meeting took place in Madrid on 3 July 2018. Every expert made an oral presentation reviewing the literature, including legal aspects if needed, related to the topic based on his/her expertise, followed by a group discussion. Presentations, scientific documents and discussions were used for the writing of this document. The event was organized by the Improvement of Health by Fitness, Nutrition, and Exercise (ImFINE) Research Group in collaboration with the Universidad Politécnica de Madrid and the Spanish Foundation for Sustainable Palm Oil.

## 2. Sustainability of Oil Palm Crops

The concept of sustainability has evolved over the years. Initially, the Brundtland report [13] defined the concept of sustainability as “meeting the needs of the present without compromising the needs of future generations.” The Food and Agriculture Organization (FAO) extended the concept of sustainability to the realm of food by defining sustainable diets as “those that generate reduced environmental impact and contribute to the food security and nutrition and to current and future generations to lead a healthy life. In addition, they protect and respect the biodiversity and ecosystems, are culturally acceptable, accessible, economically fair and affordable and nutritionally adequate, safe and healthy, and optimizing natural and human resources” [14]. Later, in 2014, the 8th report of the FAO High Level Panel of Experts (HLPE) defined a sustainable food system as a food system that ensures food security and nutrition for everyone in such a way as not to put at risk the economic, social, and environmental foundations that help provide food security and nutrition to future generations [15]. In addition, the FAO proposes the following principles of sustainability for food systems:

- Management and conservation of natural resources.
- Efficient use of natural resources for production.
- Protection of rural livelihoods.
- Improvement of equity and rural welfare.
- Building the resilience of individuals and communities.
- Establishment of accountable and effective governance mechanisms, strengthening institutions and investment.

**Figure 1:**
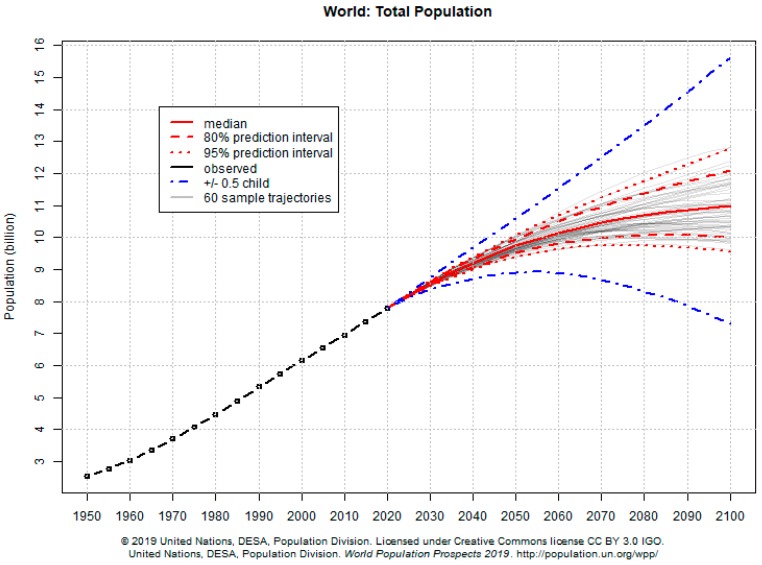
World population growth prospects [17].

In the same line, the United Nations Organization (UN) has established 17 sustainable development goals (SDGs) for the period 2015–2030, summarized in Table 1 [16]. Goal number twelve refers in particular to sustainable production and consumption, and goals two, three and six also have derivatives which affect food production and the relationship between food and health. We must not forget that the world population is expected to reach 8600 million by the year 2030, and 9800 million by 2050, which certainly poses a problem of sustainability in itself [17]. The debate over palm oil is an example of the complexity of producing food for a world population that has quadrupled in the last 100 years (1920–2019) and continues to grow, especially in developing countries (Figure 1).

The sustainability of oil palm cultivation was first addressed in 2004 with the creation of the Roundtable for Sustainable Palm Oil (RSPO). The principles of sustainable palm oil include the protection of human rights in lands where it is produced. In addition, sustainable cultivation raises the issue of protection of forests and animal life in these areas, requiring the farming practices that are best suited to enhance performance and reduce crop expansion into new areas. The concept of sustainability is therefore of special importance to finding a balance between future oil palm plantations and primary forests. Palm plantations as part of sustainability programs may be the best possible alternative in the new reality of these regions, promoting economic growth, respect for human rights and workers, improvement of communities involved in the production of sustainable palm oil, and the conservation of natural resources, as well as avoiding overexploitation to ensure responsible production now and in the future [18]. The lines of action of the RSPO are defined in 7 principles, summarized in Table 2.

In addition to the certification of sustainability, the RSPO provides for several models in the industry supply chain today: Preserved identity (PI), segregated (SG), and mass balance (MB). The PI model assures that RSPO-certified palm oil products offered to the end consumer come from a single plantation and its bases of supply, and they are physically isolated from other sources of palm oil throughout the supply chain. The SG model means that RSPO-certified palm oil products offered to the end consumer come only from RSPO-certified sources. Certified palm oils from different sources may be mixed. In the MB model, the marketing of RSPO-certified palm oil products is controlled throughout the supply chain to ensure the regulation of trade in sustainable palm oil. This model allows the mixing of RSPO-certified with non-certified oils at any point in the supply chain [19].

**Figure 2:**
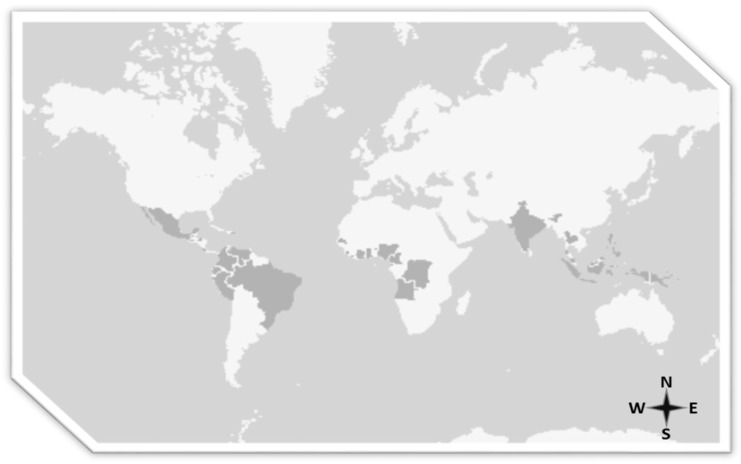
Map of palm oil producing countries (United States Department of Agriculture (USDA), 2018).

Palm oil cultivation is only possible in tropical areas where the density of primary forest is the highest in the world. Such cultivation has been brought into question because it promotes deforestation and destruction of the habitat of orangutans in Borneo. Voigt et al., say that the population of orangutans in Borneo fell by 148,500 between 1999 and 2015 because of poaching, logging, deforestation and monoculture plantations, showing that demand for natural resources itself has eliminated more than 100,000 orangutans [20]. In this connection, European Parliament resolution 2016/2222 on palm oil and deforestation of rainforests is one of the most important documents so far on the effects of the production and consumption of palm oil in the world. This document reflects the pros and cons of palm oil, but also opens the door to the use of sustainable palm oil to fight against aspects that do not comply with the principles of sustainability. The document highlights that almost half of current tropical deforestation (49%) is the result of illegal logging caused by commercial agriculture and that this destruction is driven by external demand for agricultural commodities such as palm oil, beef, soya, and timber products. Moreover, it estimates that the illegal conversion of rainforests for the purpose of commercial agriculture produces 1.47 gigatons of carbon every year, equivalent to 25% of annual emissions from fossil fuels in the EU. It should be recalled that the cultivation of palm spans several continents (Figure 2) [21].

An analysis by Bayona Rodríguez et al., noted that oil palm was one of the plants most widely exploited by agribusiness. As well as having a great potential for carbon capture, it requires little water for cultivation, characteristics that favor its sustainability. Assessments conducted in an African palm (*E. guineensis*) plantation in the Colombian municipality of Barrancabermeja found that the daily posting of carbon per hectare (Ha) was 29 kg [22]. The rate of CO_2_ fixation per kg of oil yield is quite high, and although calculated by Ha is less than that of sugar cane, with about 44 kg/Ha/day, it is well above other major crops such as corn or sugar beet. Research has highlighted the vast carbon capture potential of this type of crops, which could reduce greenhouse gas emissions and possibly help alleviate climate change. In addition, transpiration rates are not high (1.2–1.5 mm/day as against 4–4.5 mm/day for other rainforest crops) and the water requirement is low, which gainsays the notion that this plant dries the ground. In fact, in the ecosystem of palm oil cultivation (palm, soil, weed plant) 70% of the used water resources is determined by the soil transpiration and undergrowth that accompanies these crops (weed plant), and only 30% by Palm. In this connection, other authors propose measures to improve the cultivation of oil palm and make it even more sustainable [23].

In 2013, the European Palm Oil Alliance (EPOA) was founded, a business initiative to involve and educate those interested in the full story of palm oil. This is an international project working closely with active national initiatives in different European countries, providing science-based communication and creating a balanced view about nutritional aspects and sustainability of palm oil. EPOA strongly supports the adoption of 100% sustainable palm oil.

On 7 December 2015, industry, civil society and Governments signed the “Amsterdam Declaration”. This calls for the inclusion of sustainability throughout the food production chain covered by the RSPO, which has established a commitment for implementation of 100% sustainable palm oil in the European chain for 2020. Signatory countries of the Amsterdam Declaration include the United Kingdom, Germany, Italy, Denmark, Norway, and the Netherlands [24].

Spain is one of the largest importers of palm oil in the EU, although an important part is re-exported, and together with Italy it is one of the countries whose food industry imports least sustainable oil according to a report prepared by the European Sustainable Palm Oil Organization “Making sustainable palm oil the norm in Europe” [25]. This report shows that 69% of the palm oil used in Europe was sustainable in 2016. Spain, Italy, Germany, and Holland are the European countries that import most palm oil, and moreover are major distributors in the European market. This report also reveals that 60% of imported palm oil for food in Europe is officially RSPO certified (Certified Sustainable Palm Oil, CSPO). According to the “Economic Report on palm oil in Spain 2018”, in 2016, 23.7% of palm oil marketed in Spain was CSPO. This percentage rose to 29.9% in 2017, and reached 43.7% in 2018 [26].

As for palm kernel oil, in 2016 the CSPO rate was 14.9%, a percentage that remained stable in 2017, approaching 18% in 2018 [26]. This does not mean that all other palm oil or palm kernel oil is not sustainable; it only means that it is not certified as such. Nowadays, all the palm oil imported in Spain is traceable to the factory where it has been produced in the country of origin, and although it does not come with the RSPO certification, in many cases the marketing companies have their own ‘non-deforestation’, ‘No sowing in wetlands (peat-lands)’, ‘No labor exploitation’ and other such policies. In this connection there are other entities and non-governmental organizations (NGOS) such as The Forest Trust (TFT) that control the traceability of the raw material.

As regards sustainability, it is worth noting that 100% of the palm oil used for biofuels is certified by the ISCC (International Sustainability and Carbon Certification) scheme, approved by the EU through Directive 2009/28/EC on promotion of the use of energy from renewable sources [27].

Following the ESPO document, during 2016 the EU acquired a total of 7.07 Mt of palm oil, equivalent to 11% of world production, making it one of the three largest importers along with China and India. Of the total imported to the EU in 2016, 3.7 Mt went to the food industry, while the remaining 3.4 Mt went to the energy sector [25].

Today, food production systems and excess food wastage, present more than reasonable doubts as to whether the current system of food production and distribution is sustainable. According to the FAO report “Food Wastage Footprint. Impacts on Natural Resources”, 1.3 billion tons of food are wasted annually, causing great economic loss and serious damage to the natural resources on which humanity depends to feed itself. It is estimated that after the US and China, the greatest source of greenhouse gases (GHG) is the huge amount of food waste generated in the world (more than 1/3 of the total) [28]. According to Ignacio Trueba, FAO representative in Spain at the 6th AECOC meeting against food waste in Madrid in September 2018, if food waste was a country, it would be the third in the world in GHG production [29], a total of approximately 3.3 billion tons of CO_2_ per year [28]. This is also an important factor to take into account when it comes to talking about sustainability, like the water footprint, the carbon footprint, and the ecological footprint of food production.

Thanks to the work done over the last few years, palm oil is one of the leading products in sustainable production; however, there is also evident concern about sustainability in various crops globally. For example, in the case of rice, the Sustainable Rice Platform (SRP), the International Rice Research Institute (IRRI) and the UN are working on a certification that, like Fair Trade stamps or the RSPO, credit the rice planted under certain sustainable criteria. The IRRI published the standards in 2015 and is now revising the 46 base criteria, which range from use of water and fertilizers to social and labor criteria. The project also seeks to establish a compliance verification system, through audits, as a means to check on the ground that the minimum principles are fulfilled [30].

In the case of soy, the Roundtable for Responsible Soybean Production (RTRS, Round Table Responsible Soy) was created in 2009. It has a 5-year certification system based on the RTRS standard for responsible soy production and contemplates aspects similar to certificates for other crops: good farming practices, good working conditions for small producers, etc. [31]. In 2018 world soybean production was 350 Mt, of which 4,470,000 (1.28%) was RTRS certified product [32,33].

The UTZ sustainability certificate ensures that the raw material with which products are made is produced following sustainability criteria. This organism certifies, supervises and promotes improvements in farms to protect the environment, the workers and the communities where the raw materials are exploited. It currently certifies cocoa, coffee, tea, and hazelnut plantations [34].

## 3. Importance of Fats in Foods

Palm oil is included within the group of edible fats. Be it remembered that fats help to improve palatability, favor the release of flavors and aromas of meals and contribute to the feeling of satiety [35].

Sometimes maligned, partly because of its caloric content (9 kcal/g), fat is a nutrient that fulfils important functions in the organism, such as in embryonic development, or transport, metabolism and maintenance of the membrane function and integrity, cellular growth, and maintenance of tissues, brain development and vision, and transport of fat-soluble vitamins (A, D, E, K) [7,36,37,38]. Of all the FAs that make up fats, two are essential nutrients: linoleic acid of the omega-6 family (C18:2, ω-6) and alpha-linolenic acid of the omega-3 family (C18:3, ω-3). Both are precursors of other longer-chain more unsaturated FAs such as Dihomo-gamma-linolenic acid (C20:3, ω-6), Arachidonic acid (C20:4, ω-6), Eicosapentaenoic acid (C20:5, ω-3) and Docosahexaenoic acid (C:22, ω-3), precursors in turn of active biomolecules such as eicosanoids (20 carbon atoms) and docosanoids (22 carbon atoms). Eicosanoids include prostaglandins, prostacyclins, thromboxanes, leukotrienes, hydroperoxyeicosatetraenoic acid (HETE), lipoxins, or resolvins of the E series. Prominent among the docosanoids are neuroprotectins, maresins, and resolvins of the D series.

It is worth noting that depending on the FA from which they come, eicosanoids display different (even opposite) properties in terms of platelet aggregation, vasoconstriction, inflammation, and chemotaxis, among others [7,38], so that an appropriate ratio between FA ω-6 and ω-3 is essential to avoid quasi-exclusive consumption of FAs of one or other family.

Cholesterol is one of the lipids of animal origin and is a fundamental element for our organism, since it is present in the cell membranes and is a precursor of hormones (progesterone, testosterone, cortisol, aldosterone, etc.), acids and vitamin D. In general, it can be said that vegetable fat sources such as palm oil do not contain cholesterol and contain more linoleic than alpha-linolenic acid.

Body fat is the most efficient way of storing fat (less space and demand for water, and higher energy efficiency) to maintain an energy reserve. Fat surrounds the organs and bones of our body, protecting from blows and traumas, and plays an important role in the face of cold and hypoglycemia. It should not be forgotten that the composition of cooking fat very much conditions the quality of the fat in the diet we consume [7]. The recommendations for daily intake of fats are 20%–35% of the daily energy supply, which for a standard diet of 2000 Kcal is around 45–75 g/day. Rather than total fat, for which the margin of recommended intake is an ample one, scientific associations emphasize the contribution of the different FAs (saturated (SFA), monounsaturated (MUFA), and polyunsaturated (PUFA) of the ω-6 and ω-3) families [37,38].

However, these recommendations have the disadvantage of generalizing, glossing over particular aspects of great importance. Thus, not all SFAs or PUFAs have the same effect on cholesterol, platelet aggregation, or thrombogenesis. The position of the FA in the triglyceride molecule (TG) conditions its effect, so at present there are no consensus recommendations for individualized consumption of the different FAs [39]. The American Heart Association (AHA) and the FAO/WHO emphasize reduction of SFA intake (10% of total energy) [37,40], while the FESNAD stresses assurance of a high intake of MUFAs (>20% of total energy) and essential FAs, the percentage difference being made up of SFAs and all the other PUFAs [41]. This means that fat intake has to be analyzed in the context of the diet, depending on whether it is Western type (AHA, FAO/WHO) or the Mediterranean type (FESNAD) (Table 3).

### 3.1. Fat Intake in Spain

The data from the ANIBES study carried out on a representative sample of the Spanish population situate the average daily energy intake at 1810 ± 504 kcal (between 1957 ± 531 kcal for men and 1660 ± 427 kcal for women) [42]. Total fat contributes 38% of Kcal, of which MUFAs contribute 16.8%, SFAs 11.7%, PUFAs 6.6%, ω-6 FAs 5.4%, and ω-3 FAs 0.6% [42]. Fat sources in the Spanish population are mostly derived from the consumption of oils, principally olive (32%), followed by meat (22.5%), and dairy products (13.5%). In the case of SFAs, the food groups are the same, although in different order: meat (25.7%), dairy products (23.7%), and oils (21.4%) [42]. A food consumption report in Spain 2018 shows that olive oil consumption was 3.8 liters/person/year (L/p/y), milk 69.8 L/p/y, dairy derivatives 35.5 L/p/y, fresh meat 33.5 kg/p/y and processed meat 11.6 kg/p/y. Consumption of the pastries and biscuits group was 5.9 and 5.2 kg/p/y, respectively [43].

Recent publications have shown there is a lack of data regarding palm oil intake in Europe and Spain [3,39]. It is necessary to contextualize the content of palm oil in foodstuffs and a Mediterranean-type diet. The palm oil present in our diet comes from products located at the top of the food pyramid, so-called indulgence foods, which should only be consumed occasionally. The current problem is that despite the decrease in calorie consumption, the diet is saturated by an increase in the frequency of consumption of indulgence products. The excess energy induced by a positive energy balance presents a new scenario in the interpretation of health and nutrition. Physical activity exerts a regulatory effect on the energy balance greater than the caloric intake [44]. Physical inactivity promotes a constant positive energy balance, and in the Spanish population, sedentary rates exceed 70% and more than one third of the population fails to comply with the minimum recommendations of daily physical activity [45,46].

### 3.2. Complexity of Fat Metabolism

When assessing what fraction of the food we ingest is absorbed in the organism and subsequently metabolized, it is essential to consider the concepts of bioaccessibility and bioavailability. Bioaccessibility means the maximum fraction of the nutrient that can be released from the food matrix in the gastrointestinal tract, while bioavailability is the fraction that reaches systemic circulation from the gastrointestinal tract and which is available to promote its action within the organism.

The majority of the fats in foodstuffs are composed of TGs, which need to be hydrolyzed into FAs and glycerol before being absorbed. The disposition of the FAs that are attached to the skeleton of the glycerol in the TG molecule generates a stereospecific molecule that determines its absorption. Pancreatic lipase hydrolyses positions sn-1 and sn-3 FA, generating 2-monoacylglycerides (2-MAG) and free FAs (FFAs). In the presence of divalent ions (e.g., calcium), the SFAs released from positions sn-1 and sn-3 form insoluble soaps that are not absorbed. This singularity is lost when the SFAs are in the sn-2 position of the TG. FFAs and MAGs are absorbed directly by diffusion in the enterocytes of the intestinal wall. The phospholipids are hydrolyzed by the phospholipase A2, producing FFAs and lysophospholipids. FAs with chain lengths greater than 14 carbon atoms are re-esterified in the enterocyte and are circulated through the lymphatic pathway in the form of chylomicrons. Short and medium chain FAs go directly to the liver through the portal vein. Cholesterol esters are hydrolyzed by pancreatic hydrolase of cholesterol esters. Fat-soluble vitamins and cholesterol also reach the systemic circulation incorporated in the chylomicrons.

This complicates the study of fats in general, and palm oil in particular, in that such studies need to take into account not only FA composition but also positioning within the TG.

## 4. Nutritional Composition of Palm Oil

The FA balance of palm oil is around 48% SFA and 52% unsaturated (UFA)—that is, in terms of SFA values, higher than other oils such as sunflower, olive, or rapeseed which are classified as healthy, and well below coconut oil, which has a high SFA content, mainly myristic and lauric. Palmitic acid is the most abundant of palm oil SFAs, reaching 85%. And for its part oleic acid is the largest UFA component (88%) in palm oil. In addition, the content of FAs such as lauric acid (12 atoms of C) and myristic acid (14 atoms of C) accounts for 1.5% of SFAs, which is very minor compared to coconut oil.

In palm oil, 80% of the oleic acid is located at the sn-2 position of the TG, so it is quickly absorbed as MAG. Based on the stereospecificity of the positioning of the FA in the TG and the bioavailability of the FA in sn-2, mainly oleic acid, palm oil may be considered to act as a MUFA on the plasma lipid profile [47,48].

Palm oil is more complex than simply the oil *per se*, as the composition of the different fractions currently marketed and used by the industry depart from the standard 50%/50% profile. In this context, palm olein contains 44% SFA and 56% UFA. Its characteristics make it more liquid, and it is normally used in the industry for potato crisps, etc. At the other end of the spectrum is the palm stearin fraction, with a higher SFA content (68%) and a lower UFA content (32%), which is used to make products such as croissants that require more solidity, and is an alternative to the use of hydrogenated oils, so reducing the intake of trans-FAs (Table 4).

**Figure 3:**
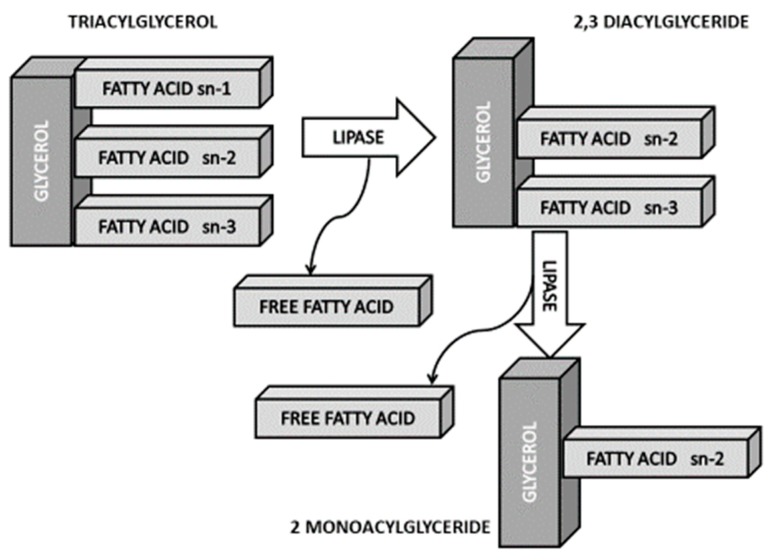
sn-2 hypothesis. Modified from Gesteiro et al. (2018) [39].

The percentage of SFAs is generally higher in animal fats (meat, dairy), so the replacement of animal by vegetable fats appears a favorable alternative. The debate currently focuses on the FA composition of vegetable oils, especially palm oil. When comparing palm oil with fats of animal origin that have a FA profile similar to lard and are less saturated than butter, be it noted that since an important part of the palm oil SFAs are in position sn-1 and sn-3, SFA absorption is lower and hence its potentially negative effects for cardiovascular health are also lower (Figure 3). It is worth noting that in addition to these stereospecific differences, palm oil differs from butter in that it contains less butyric (not detectable vs. 3.32%) and stearic acids (3.5%–6% vs. 9%), and more oleic (36%–44% vs. 22%) and linoleic (9%–12% vs. 1.1%) acids [49,50,51].

Palm is one of the most stable oils, prolonging food storability. The resistance of products with palm oil to rancidity derives not only from its SFA content, but also from its antioxidant components (mainly tocotrienols and tocopherols), thus avoiding lipid peroxidation and the consequent formation of free radicals. However, during the refining process, the antioxidant content of palm oil is reduced dramatically. In Spain only refined palm oil is permitted [4].

### Palmitic Acid

One of the arguments underpinning the questioning of palm oil healthfulness is its SFA content, and specifically the relationship of palmitic acid to cardiovascular health [52,53]. Palm oil is often mistakenly identified with palmitic acid, and we therefore consider it necessary to look more closely at the nutritional and metabolic aspects of this FA. The first thing to emphasize is that palmitic acid is a long chain SFA (16 carbons), and not only is not itself a toxic compound, but is synthesized endogenously in the organism; in fact, it is the most abundant FA in the human body, accounting for between 20%–30% of total FAs. It is an essential component of cell membranes, secretory lipids, and transport and is part of the normal composition of the human organism [54]. It is found especially in fetus fats (45%–50% of FAs) and in breast milk [54,55]. It has other functions in addition to the production of energy, such as the ability to join certain proteins necessary for some functions of the nervous system, to promote the formation of the pulmonary surfactant or to guarantee the binding between cells [56].

The richest foods in palmitic acid are dairy products, meats (fatty cuts of red meat and poultry skin, such as chicken) and vegetable oils (particularly palm and coconut). Human milk is richer in palmitic acid than cow’s milk. In fact, it is the most abundant FA in the composition of that milk, accounting for between 20% and 25% of the FAs. Like most animal fats, palmitic acid is mainly located in the sn-2 position of the TG, in such a way that promotes its absorption. Palm oil is used as an ingredient in follow-on milks and infant milks, and should be interesterified, since in palm oil, palmitic acid does not preferentially occupy the sn-2 position.

A recent review of opinion delves into the effect of diet and the synthesis of endogenous palmitic acid through de novo metabolic pathway lipogenesis, focusing attention on the importance of a balanced UFA/SFA ratio in the phospholipid membrane, and especially emphasizing the role of the PUFAs in this balance [56]. From a metabolic point of view, an imbalance in the SFA/UFA ratio can promote de novo lipogenesis and cholesterol synthesis and moreover, the replacement of SFAs by PUFAs can induce an increase in the palmitic acid levels in the tissue through de novo lipogenesis. The endogenous synthesis of palmitic acid is also reviewed, explaining the mechanisms that can potentially intensify this process, such as an excess of energy in the diet. And again, it relates de novo lipogenesis to the emergence of cancer, in that this is a preferential metabolic pathway in most tumors and explains this process at the molecular level. It stresses the intake of nutrients through the SFA/PUFA balance; it explains the CD36 FA receptor and the role of palmitic acid in its activation, mentioning the study by Pascual et al., [57], but it also raises the need to study the behavior of CD36 as a receptor in the intake of other sources of energy, such as other FAs or carbohydrate-rich diets. The conclusion is that under normal physiological conditions, it is de novo lipogenesis which regulates the accumulation of palmitic acid in tissues; however, in situations such as positive energy balance or inactivity/sedentarism, mechanisms responsible for maintaining equilibrium in the concentrations of palmitic acid in the tissues are interrupted, causing dyslipidemia, hyperglycemia, fat accumulation and inflammation.

Authors should discuss the results and how they can be interpreted in perspective of previous studies and of the working hypotheses. The findings and their implications should be discussed in the broadest context possible. Future research directions may also be highlighted.

## 5. Palm Oil and Its Effects on the Lipid Profile

The relation of palm oil with CVD has been widely studied [7,58,59], and there are numerous systematic reviews and meta-analyses in this respect [52,60,61,62].

The results reported by Temme et al., suggest that medium chain FAs (between 6 and 12 carbons) raise the TG level, but have little effect on TC and HDLc levels [58]. In addition, they do not induce lipogenesis and are quickly used up for energy purposes by means of beta-oxidation, unlike long chain FAs, particularly all the SFAs that are stored through high intake, promoting obesity [7].

Some authors have related palm oil intake to cardiovascular health problems [59], but recent reviews indicate that there is no real scientific evidence for it [61,63]. It is worth noting that in the countries where this oil is used for frying, the number of deaths attributable to this pathology is no higher than in importing countries (Malaysia 35%, Indonesia 35%, Germany 37%, Spain and Belgium 28%, and USA 30%) [64]. This suggests that it is not so much the oil that is used, as the diet as a whole, as well as lifestyle. In fact, high SFA intake in the context of a hypercaloric diet induces insulin resistance and less satiety than in conditions of limited intake and low fat intake, when the effects are lesser [65].

The reviews by Fattore et al., and Sun et al., show an increase of LDLc and TC with age and consumption of diets rich in palm oil [52,60]. Recently, Gesteiro et al., analyzed these studies in depth, observing discrepancies when defining the FA profile of the oils used in the plasma lipid profiles of the populations studied, as well as the oil considered as an ingredient or dressing. Physical activity, often forgotten in nutrition studies, is another of the variables that have not been considered in most studies [39].

Recent reviews of studies on the effect of palm oil intake on plasma lipid profile have observed that many of these studies have not been taken into account when analyzing results for the position of the FA in the TG [48,66,67,68]. Another little-studied but no less important aspect is the effect that the most saturated oils used in the studies exert on lipoprotein oxidation. Some authors suggest that this type of research should include the lipoprotein used in biomarkers of CVR in foals, such as apolipoproteins (APO) APO A1 and APO B (some linked to specific polymorphisms). In this respect, few studies have gone further into the effects of palm oil consumption or its fractions. Some authors posit a raising of Apo A1 levels [52]. Cuesta et al., studied the effects of consumption of a palm olein on high oleic sunflower oil in a controlled population of postmenopausal women. This intervention consisted in replacing 9% of the energy in the diet derived from oleic acid with palmitic acid (approximately 18 g of total SFA or 18 g palmitic acid). There were considerable increases of both LDLc (21.6%) and HDLc (14,9%), but LDLs were less oxidized (70% less peroxides); this was a consequence, among other aspects, of very significant (*p* < 0,001) beta-carotene, (All-E)-beta-carotene and cryptoxanthin demand, although there was a decrease of lycopene [69]. This is an important aspect in that oxidized LDLs and not LDLc are decisive in initiating the atherogenic process [70,71]. They also observed a non-significant reduction (3%) in APO A1 levels, with a higher APO B level (11.8%) and a reduced APO A1/APO B ratio (22.8%), suggesting an increase in CVD risk after consumption of some palm oil fractions. However, no significant changes were found in other very predictive markers of CVR such as the quotient of total cholesterol (TC)/HDLc or LDLc/HDLc. Subsequently, Sánchez-Muniz et al., observed that the same dietary change induces a very significant increase in the HDL APO A2/APO A1 ratio [72]. Since the increase in APO A2 levels is considered a limiting factor in the esterification of cholesterol via lecithin cholesterol acyl transferase (LCAT), retrograde cholesterol transport and antiatherogenic capacity of HDL [73], this change is considered non-beneficial. The exchange of the two oils also induced rises in thromboxane A2 levels (TXA2, measured as thromboxane B2), with platelet activity, and to a lesser extent also prostacyclin I2 (PGI2). The net change involved increases in the antiphospholipid TXA2/PGI2 index, suggesting a prothrombogenic effect post-intervention [74].

Manipulating the fat in the diet can have beneficial effects in terms of both quantity and composition, although the response is not homogeneous throughout the population, in which individuals have been defined as normo-, hyper-, and hyporesponders. In fact, in studies where high oleic sunflower oil was replaced with palm olein in postmenopausal women, as noted above, the most important differences in the oxidized LDL, APO A2 and APO A1/APO A2 ratios were found in women with hypercholesterolemia, with no major differences in other markers between normo- and hypercholesterolemia. This difference in response is linked to nutrigenetic mechanisms that relate to the presence of risk alleles in certain candidate genes [75,76].

In a recent double-blind, cross-over randomized intervention study conducted in China, comparing the daily intake of 48 g of olive oil with 48 g of palm oil in 120 adults for 2 months with a washout period of 2 weeks, no differences in the effect on body fat and plasma lipid profile were observed [77].

Whereas trans-FAs act to reduce levels of HDLc, palm oil has been shown to increase HDLc values. In addition, the increase in LDLc is significantly greater when trans-FAs are consumed rather than palm oil. When a vegetable fat (palm oil) is compared to an animal fat (lard), there are also improvements in the lipid profile, despite the FA composition being similar in some cases. These data support the theory cited above as to the importance of FAs in the sn-2 position. Hence, with what we know to date, it is fair to say that vegetable fats (in this case palm oil) constitute a healthier alternative for lipid profiles than trans-FAs [52,60].

Schwingshackl et al., recently published an interesting meta-analysis whose main objective was to analyze the effect of a series of 13 fats and oils on LDLc levels, including a secondary analysis of the effects on TC, HDLc, and TG. To do this, they analyzed 54 studies published between 1984 and 2018, with a total population of 2065 participants. All of them were randomized trials, comparing at least two of the 13 oils and fats contemplated. TC, LDLc, HDLc, and TG were analyzed for the plasma lipid profile. Safflower, rapeseed, flaxseed, sunflower, corn, olive, soybean, palm, and coconut oils, and beef fat, were the ones that most reduced LDLc and TC when used to replace 10% of the dietary energy intake from butter. Safflower, sunflower, rapeseed and corn reduced TC much more than coconut or palm, while safflower, sunflower and rapeseed were more effective than olive oil in reducing TC. The replacement of 10% of the energy from butter with the equivalent in sunflower, palm, olive or coconut oil, raised HDLc concentrations. Coconut and palm oil raised HDLc more than corn or soybean. In the case of TG, the oils that most effectively reduced plasma concentrations were sunflower, soybean, and palm. When comparing oils with beef fat, the ones that most reduced TG plasma concentrations were safflower, sunflower, corn, soybean, and palm. Palm oil was found to be more effective in improving HDLc than oils with lower SFA content; and when compared with UFA-rich oils, it increased both plasma LDLc and HDLc concentrations. The quality of the evidence was moderate for TC and poor or very poor for LDLc. In the case of HDLc and TG, the evidence turned out to be moderate [62]. The explanation for this low evidential level may include the small number of studies analyzed, risks of error, inaccuracies and inconsistency of some comparisons. It is striking that the authors did not consider cocoa butter, which is rich in SFAs and is widely used. In the case of palm oil, a standard FA composition was considered, which can cause errors, since the behavior of the different palm oil fractions differs according to the cardiovascular profile. The time interval considered is very long, and this should be taken into account, since the analytical techniques used to determine the different cardiovascular profile markers have evolved a lot during the 34 years covered by the review and this may not have been taken properly into account.

The authors of a recent review, which includes four studies on coronary heart disease and palm oil (all conducted in Costa Rica and belonging to the same research over time), and one that analyses the association of accident stroke with palm oil, called attention to a few studies included in the review after applying the PRISMA methodology (Presentation of Systematic reviews and Meta-Analysis). Although they found an association between palmitic acid and the risk of myocardial infarction, there was no such association with total SFA intake. The authors insist that many factors recognized in relation to CVR, such as hypertension, diabetes, alcohol use, lack of physical activity, tobacco, etc., are responsible for 90% of the population attributable risk. They also suggest that instead of considering nutrients in isolation, the diet should be analyzed as a whole. It is evident that research of greater scientific and technical quality is needed in relation to the consumption of palm oil and CVR. In the conclusions, which according to the authors should be considered with caution, they indicated that there is no evidence of an association between the consumption of palm oil and risk of death from CVD. Likewise, they pointed out some needs identified in relation to research in palm oil, such as (1) to assess the consumption of palm oil quantitatively and with greater rigor; (2) to analyze the consumption of palm oil independently of other foods; (3) to conduct studies of sufficient depth and duration; (4) to adjust to the known CVR factors when performing statistical analysis; and (5) to include countries with high palm oil consumption in the studies. This review highlights the difficulty in establishing differences between types of fats, FA sources, and the effects of replacing some fats with one another, since they depend on the dietary context where the study is conducted and on the research design [61].

In preparing reviews or meta-analyses with nutritional studies, several difficulties arise when making estimates of the composition, such as differences in the composition of food over the years, state or companies’ own reformulation programs, or labelling regulations. Faced with this situation, the question is how extensive are the studies that have been published so far, or if the results are extrapolated to the present day taking these possible variations into account. In studies of cardiovascular disease (CVD) of only a few years ago, the biomarkers that were used were HDLc/LDLc, in addition to TC and TG. Moreover, different HDL fractions (HDL_1_, HDL_2_, HDL_3_) and the sizes and density of LDL were studied to determine their degree of atherogenicity. In reviews and studies on palm oil, these biomarkers are not presented. Unfortunately, and quite possibly due to financial constraints, both in clinical trials and in many research projects, the parameters studied are almost exclusively TC, TG, LDLc, and HDLc, ignoring the prognostic value of other protective markers and CVR.

Some reviews and published meta-analyses are summarized in Table 5.

### Palm Oil and Microbiota

It is well known that the consumption of dietary fiber and the variety of the same is the factor that most influences the quality of the intestinal microbiota, but until recently the impact of dietary fats on it was unknown. Research such as that of Kübeck et al., or Just et al., have begun to shed some light on the matter in animal models [85,86].

The research results of Just et al., indicate that the intestinal microbiota modulates the lipid profile, and that the impact of dietary fat, whether of plant or animal origin, on the lipid profile depends on the microbiota. At the same time, dietary fat modulates the microbiota present in mice, so that the microbiota of mice which followed a low-fat diet were dominated by two lactic species, producing short-chain (acetate, propionate, butyrate, and isovalerate) FAs, while high-fat diets altered the metabolic capacity of amino acids, as well as the functional capacity of the microbiota. Mice fed with a fatty diet of palm fat were the ones that developed a greater number of peptide similar to glucagon-1 (GLP-1) enteroendocrine cells, which could account for better glucose tolerance in these animals [86].

## 6. Palm Oil in Animal Nutrition

Diet is a determining factor in the composition of products obtained from monogastric animals (birds and pigs). On the other hand, in ruminants (bovine, ovine and caprine), dietary fats are not directly reflected in the composition of the fat of the animal, since the ruminant incorporates the products of the transformation of the fat in the rumen. The digestive cavity in ruminants is divided into four compartments: reticle, rumen, omasum, and abomasum (in this order in the cranial–caudal direction). The conditions there—anaerobiosis, aqueous medium, high temperature, and a wide presence of microorganisms (bacteria, protozoa, and fungi)—cause it to act like a fermentation chamber.

The main energy ingredients in feed for ruminants are cereals, followed by vegetable oils (palm, soy, and sunflower), animal fats (lard and tallow) and industrial greases (inert greases such as soaps and hydrogenated palm oil and oleins). In suitable proportions, these fats and oils improve the energy density of their diets. However, vegetable oils containing a high concentration of UFAs, interfere in the progress of ruminal fermentations, inhibiting microbial activity. Therefore, ruminants hydrogenate fats to protect themselves from the negative effects that UFAs have on them, thus engendering the natural trans-FAs present in products from ruminants.

Thus, while in compound feed intended for monogastric animals, the type of fat used is unsaturated or a combination of saturated and unsaturated (e.g., high oleic sunflower for Iberian pork fat and butter or soy for birds), in food for ruminants saturated fat is preferable. Consequently, of the vegetable oils available in the marketplace, palm oil is the one most used in ruminant diets. This is because it contains more SFAs and hence has less of an adverse effect on ruminal microbiota, and a much more competitive and stable price on the commodity market than other vegetable oils.

In 2017, in the EU-28 159 Mt of compound feed was produced for livestock, where the addition of ingredients such as oils and fats accounted for 2% of output [87]. In 2017, more than 35 Mt of feedstuffs, of which nearly 10 Mt were for ruminant animals, were manufactured in Spain [88].

Consumers nowadays demand good quality products at a low cost. The production of meat products to meet demand requires compound feed to ensure food safety and a balance in the price of the final product. In the current food composition tables, compared to 30 years ago, the fat content of foods of animal origin has decreased a lot (around 15%). Meat, other than offal, contains 4%–5% of fat [50].

## 7. The Use of Palm Oil in the Food Industry

To contextualize palm oil and its use in the food industry we must briefly analyze the evolution in the use of SFAs by the industry. Thanks to their consistency, SFA-rich fats provide the appropriate texture for a series of foods both in traditional domestic use and in the industry. These textures are not achieved with less SFA-rich oils or fats. Traditionally, animal fats were used for their palatability and experience in consumer tastes. As scientific studies indicated that animal fats with higher SFA contents are responsible for CVD, these were replaced by hydrogenated vegetable oils (also solids), offering great versatility in terms of textures, with a consistency similar to animal fats and containing trans- FAs. In the 1990s, the first lipid profile studies on the effect of trans-FA consumption revealed the negative effect of this type of fat on lipid biomarkers and the CVR associated with its consumption [6]. Initially, the labelling of such products was regulated, making it obligatory to distinguish between oil and fat, animal and vegetable, as well as totally or partially hydrogenated fats, so that the consumer ended up associating the word “hydrogenated” with “*trans*” and with unhealthy. Scientific evidence has now prompted regularization, with prohibition of the use of “*trans*” in some European countries such as Denmark, the Netherlands and the USA. Vegetable fats and oils have replaced the use of trans-FA-rich hydrogenated fats almost entirely in the European industry. The Spanish Food Safety and Nutrition Act deals with trans-FAs, stating that “in industrial processes in which trans-FAs can be generated, the responsible operators will establish adequate conditions to minimize the formation of these where they are intended for food, either as such or as a food component. In addition, they will ask their suppliers for information on the trans-FA content of the food or raw materials they provide and will have at their disposal the information related to the FA content in their products. These requirements will not apply to products of animal origin that naturally contain trans-FAs”. As indicated in the introduction of this document, both the WHO and scientific societies recommend that the intake of trans-FAs be less than 1% of the total daily energy intake [89]. In 2015, the European Commission issued a report urging that trans-FA content be reduced as much as possible [90]. EU Regulation 2019/649 specifies that the content of trans fats, in foods intended for the final consumer and supply to retailers, should not exceed 2 g per 100 g of fat, provided that trans fats are not naturally present in fats of animal origin [91]. Thus, the Commission responded to resolution 2016/2637 in which the European Parliament requested the establishment of a legal trans-FA limit for the entire EU [92].

### 7.1. Justification for the Inclusion of Palm Oil in the Composition of Processed Foods

Palm oil is used for various purposes in the food industry: Frying, margarines (puff pastries, croissants), toppings and fillings, and others (premixes, emulsifiers). Among the technological characteristics that it contributes is a melting point close to 37 °C, guaranteeing a product with some solidity that melts on the palate when consumed, producing an effect that is not achieved with any other oil. In addition to its palatability and structure, the great stability of this oil in the frying process obviates the need to use antioxidant food additives. Another outstanding factor is the versatility of palm oil, in that different fractions can be mixed to achieve different strengths and textures, such as croissants and puff pastries, unattainable with other oils or fats. If we take ice creams as an example, at first these were made with milk fat. Little by little this was replaced by coconut fat, which a few years later was replaced by palm fat, whose price was more competitive. In recent years, some manufacturers have been turning to coconut fat because the use of palm oil is being questioned. It should be noted that coconut oil has a more atherogenic FA profile than palm oil, mainly due to its high content of medium chain SFAs (lauric and myristic). Other candidates to replace palm oil in the industry include: shea butter, with an annual production of 400,000 tons (75% for internal consumption in the countries of origin); cocoa butter, production of which reaches 1 Mt per year; butter, with very low availability; and hydrogenated seed oils. None present technological advantages comparable to palm oil, and all are much more expensive. European Regulation 1169/2011 on food information provided to the consumer establishes that both vegetable oils and refined vegetable fats, if they have been subjected to hydrogenation processes, must be labelled as “totally hydrogenated”, or “partially hydrogenated”, as the case may be, guaranteeing less than 1% of trans-FAs [93]. Up to 60 Mt of palm oil is produced per year, and hence it maintains a lower and more stable price in the vegetable fat market. All the above-mentioned candidates to replace palm oil (cocoa, shea butter, butter) suffer from high price volatility and low availability, which encourages the industry to lean towards palm oil.

Although of potential interest from a nutritional point of view because of its high tocotrienol content, the use of red (crude) palm oil is ruled out because of its variability, acidity, color, and the flavor that it would impart to the food product, altering the consumer’s perception of palatability. The Spanish quality standard for heated oils and fats establishes a maximum of 20%–25% polar compounds in frying oils [94]. Palm oil leaves 8% of these compounds in the form of diglycerides (DG), but as it is more stable due to its high SFA content, it takes longer to reach the limit values.

Studies by the Sánchez-Muniz group with the palm olein fraction have demonstrated the enormous stability of this oil in repeated potato fries, even when the oil is not renewed [95]. However, given its high DG content palm olein may not be suitable for frying certain foods. But at the same time there is a perceived need for the food industry to find a balance between the negative aspects for cardiovascular health derived from the SFA component and the positive aspects of consumption of low levels of oxidized and polymerized FAs [96]. The health effects of altered FA consumption described so far derive from studies in which alteration components were isolated in high doses or from studies with highly altered oils and remote from daily consumption rates. Frying temperatures above 200 °C considerably promote the dimerization and polymerization of TGs. Dimers and polymers are the most toxic compounds, although they tend to be less absorbed. The results suggest that the consumption of altered oils with high concentrations of free radicals and polymerization compounds could raise the CVR and susceptibility to damage in membrane lipoproteins and affectations of the hepatic microsomal system via CYP4A1 and the regulation of genes dependent on the transcription factor PPAR-alpha [97,98] for although they are partially neutralized, they are absorbed and metabolized [96,97]. These authors have indicated that intake of highly thermo-oxidized oils reduces intestinal antioxidant defense in rats, modifying the gene expression of antioxidant enzymes, and the damage is greater when the oils are consumed after a 15-hour fast period [99]. However, again we must insist that much information relating to altered oils and toxicity derives from the study of oils subjected to excessive heating (not oils used in frying) and that the frying of food can be considered a safe technique when the oils are used for limited periods of time, correctly, at controlled temperatures, and under regulated oil renewal conditions [97,98].

### 7.2. Role of Industry in Sustainable Palm Oil

As already mentioned above, solid fats at room temperature and with a certain degree of hardness, such as butter, margarine, and frying oils, are essential in the preparation of some foods. Until the producing companies opted for the non-use of partially hydrogenated oils, palm oil was a component in the fats prepared for the food industry. Since then the use of palm oil has increased, either as such (100% palm), or mixing something harder with palm fractions. Some producers also occasionally use interesterification with mixtures of other oils to achieve a smoother solids curve and reduce the difference between the solid and the liquid phases. Currently, palm oil in its various forms is therefore the only economically viable alternative for use in certain applications as a substitute for trans-FAs.

As a result, distribution requirements have turned on food security (minimization of the presence of chemical contaminants, technological aspects), and on sustainability (mainly in RSPO certification), initially in the MB form and a second stage, in the SG form, which entails a great deal of control and traceability of the distribution chain. The advantage of the RSPO certification is that the consumer can read the guarantee of sustainability on the label.

As already mentioned above, one of the qualities of palm in frying oil is its high stability. This means that in fryers operating continuously with a rate of renewal (turn-over) less than 10 h, there is no need to change the oil, only to replace the quantity absorbed by the food. Apart from oxidation stability, palm oil brings structure to the product, in such a way that when it cools to room temperature, the solid fraction provides consistency and maintains a palatable mouthfeel, avoiding the typical oily texture of products fried in vegetable oils that are liquid at room temperature.

**Figure 4:**
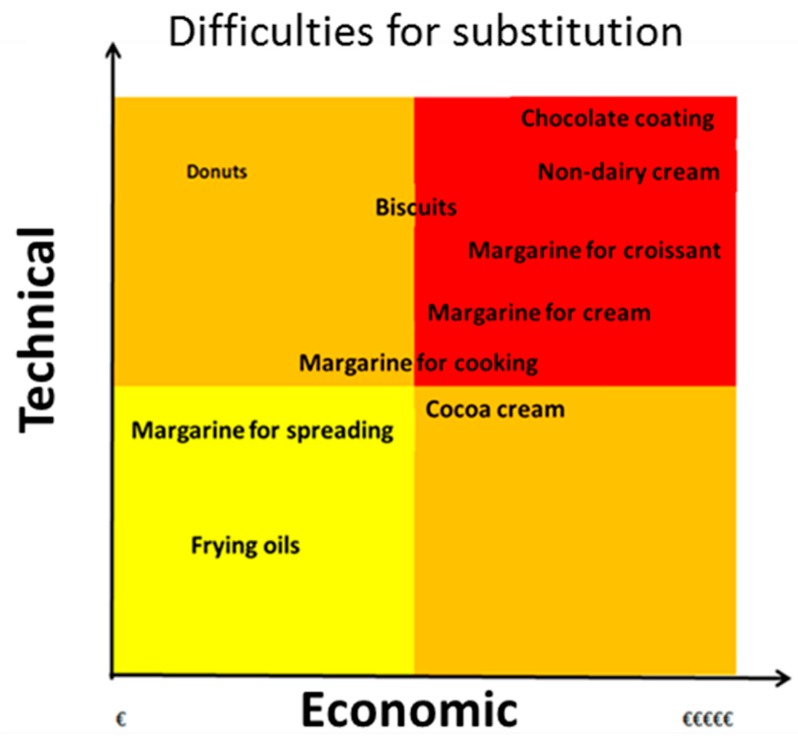
Technological and economic difficulties in replacing palm oil.

Figure 4 shows the technological difficulties and the economic cost of replacing palm oil with other fats. Palm oil is also used for the manufacture of emulsifiers; whose emulsifying capacity depends on its FA stereo configuration. For some specific applications, such as delaying aging of bread, it is important that the FA has linear configuration, as in the SFA.

## 8. Food Safety and Toxicological Aspects

Doubts have been raised about the safety of palm oil by the finding that it may contain 3-monochloropropane-1, 2-diol (3-MCPD), 2-monochloropropane-1, 2-diol (2-MCPD), glycidol and its glycidyl esters (GE), and that their presence can be detected in many foods containing palm oil [100,101]. In 2016, two scientific committees responsible for risk assessment, the JECFA (Joint FAO/WHO Expert Committees on Food Additives), and the FAO/WHO and EFSA-CONTAM-dependent (Panel of Contaminants in the Food Chain), issued two separate scientific opinions regarding toxic contaminants in palm oil and other vegetable oils which are generated in the process of refining (GE, 2-MCPD, 3-MCPD) [102,103]. Both committees carried out an evaluation of the exposure of the population to these substances through national consumption surveys, and both concluded that younger populations are most exposed. According to the EFSA and the JECFA, the main sources of exposure to 2-MCPD, 3-MCPD, and GE in the population aged 3 or over are margarine, pastry, and cakes, albeit there may be variations depending on the oil source. After analyzing the different available toxicological tests, each established recommendations for exposure to these contaminants once a health-based guide was established (Health Based Guidance Value—HBGV) for 3-MCPD and the acceptable margin of exposure (MoE) to glycidyl. Table 6 shows the average contents of these contaminants in different oils.

There are various different stages in the assessment of contaminant risk: (1) hazard identification (identifying the adverse effects on health, the target organ and critical toxicological effect (end point) of the target contaminants); (2) characterization: danger (aimed at deriving a HBGV, for example the tolerable daily intake (TDI) from one point of reference obtained from toxicological studies); (3) exposure assessment (takes into account food consumption and the emergence of chemical contaminants in food by food groups, groups of population, in acute vs. chronic exposure); and (4) risk characterization (takes into account exposure to the contaminant vs. HBGV, in this case the TDI—this is considered the intake level that poses no appreciable risk for health). The TDI provides a HBGV for chronic exposures or long-term contaminants in foods and is often established from a starting point (P0D), for example, a NOAEL (no observed adverse effect level) identified from toxicological tests to doses in experimental animals. This TDI is then compared with chronic dietary exposure.

For characterization of the hazard information, the critical toxicological points obtained from the studies conducted in laboratory animals or rarely in humans need to be established. This information will be taken for the identification of a NOAEL or similar, such as the BMDL_10_, (benchmark dose level) and thus establish a TDI.

The analysis of the “reference dose” (benchmark dose BMD) uses the average of the model resulting in the “reference dose level” (BMDL_10_); the value produced by the study is the critical point that is associated with an additional risk of 10% of the adverse effects on experimental animals exposed.

### 8.1. Definition, Formation, and Possibility of Reduction of the Contaminants Free and Esterified 2-MCPD and 3-MCPD and Glycidil Esters (GE).

These contaminants appear during the refining phase of vegetable oils, such as olive, sunflower, and palm, entailing high temperatures, especially at the deodorization stage.

The 3-MCPDs are formed in the refining of oils as well as during processing or preparation of food when exposed to high temperatures (>230 °C) in the presence of chlorine, resulting in the formation of HCl reagent. This chlorine may originate in: (a) inorganic chlorine present in fertilizers (NH_4_Cl, KCl) and flocculants used in treatment of wastewater (FeCl_3_); (b) organochlorine products generated endogenously by the oil palm; (c) chlorinated molecules which accumulate in the fruits of the palm during its growth; or day) thermal degradation causing the formation of HCl reagent during refining. Washing fresh fruit with water clusters has been found to remove 95% of the chlorinated substances [105]. Regarding 2-MCPDs, there is too little available information to be able to judge, and it is extrapolated from what is known of the 3-MCPD.

GEs are found only in oils and refined vegetable fats. They arise when high temperatures are reached in the oil deodorization process (>200 °C according to the EFSA [107], >240 °C according to the JECFA [102]), and from the transformation that occurs from the diacylglycerol, without the need of HCl.

In these contaminants, free forms are more active in vivo than esterified forms. The conversion from ester form to free form occurs through hydrolysis; complete hydrolysis is assumed in vivo and is considered also to occur in neonates.

These contaminants from processed vegetable oils have been found in greater quantity in palm oil/fat. Wong et al., have studied the formation of GEs and 3-MCPDEs in potato crisps with RBD palm olein (refined, bleached and deodorized) in a deep fryer. To this end, they analyzed the oil after frying potatoes at different temperatures (160 °C or 180 °C) with added salt (0%, 1%, 3%, and 5%) for 100 min/day over 5 consecutive days. These researchers conclude that the duration of the frying, the temperature and the concentration of salt, all play significant parts in formation of the toxic contaminant compounds referred to here. The order of the effects was: temperature > time > salt. In general, the tendency was for 3-MCPDEs to decrease with longer frying, and to increase with increasing temperature and salt concentration. The trend for GEs was to increase with the increasing of all three parameters in the study. Evaluation of the quality of the oil showed that this did not exceed the safety limits (conjugated p-anisidine, FFA, dienes and trienes), but the authors did point out the need to control the frying time and temperature, as well as the addition of salt, to foods cooked in this way [108].

Most palm oil products found on the market come in RBD forms. Refining is necessary to eliminate impurities and contaminants that affect the quality of the final product. However, it is important that the refining process retain as many natural antioxidants as possible (tocopherols and tocotrienols), in order to maintain the stability of the oil. Most crude palm oil is refined by physical processes, although a small percentage is chemically refined. Some refinement stages are performed at high temperatures (250–270 °C), which favor the formation of these toxic contaminants [109]. Other methods have been developed which in addition to conserving carotenes, are performed at lower temperatures (150–170 °C), thus greatly reducing the formation of contaminants [110].

Authors such as Willits propose measures to reduce the contaminants in oils, specifically in palm oil. RBD palm oil may have less than 2 mg/kg of 3-MCPDE or GE, if: (a) the quality of palm oil is good (low acyl-glycerol content and low chlorinated precursors; (b) natural bleaching soil is used (for low 3-MCPDE content); (c) deodorization is carried out at a temperature below 230 °C (for low GE content). In addition, there are other refining options: (a) chemical refining, with the drawback that a soapy paste can form as a lateral current; (b) physical semi-refining, in which the challenge is to have low FFA levels and a light color; (c) In any case, the heat load during deodorization has to be low [105].

### 8.2. Risk Assessment of Contaminants

In 2013, in its preliminary assessment of the risk of the presence of 3-MCPDs in foodstuffs, the EFSA estimated a mean exposure <1 µg/kg body weight/day (µg/kg bw/d), and concluded that the exposure value for the majority of the population groups was lower than the TDI of 2 µg/kg bw/d [107]. However, the publication of a second opinion in May 2016 modified these preliminary results in response to data from a larger number of samples proposing a new, lower TDI value (0.8 µg/kg bw/d). The EFSA calculated a level of exposure for 3-MCPDs and its esters exceeding the TDI in the population group of infants and children [103]. Shortly after, in November 2016, the JECFA published the conclusions of the current evaluation of 3-MCPDs and glycidol and its esters, setting a new provisional TDI of 4 µg/kg bw/d for the sum of 3-MCPD and esters [102], five-fold higher than the one established by the EFSA in its second assessment. Due to the large difference between the toxicological reference values proposed by the two scientific committees, the EFSA decided to review its scientific opinion of 2016 once again. This review of the evaluation of the risk of 3-MCPD and esters was published in 2018, setting the TDI at 2 µg/kg bw/d, the same as in its first assessment of 2013 [111]. The differences in the TDI values set by the FAO/WHO and the EFSA make it difficult to arrive at a conclusion on the TDI derived from the presence of chemical contaminants generated during refining. There is clearly a real possibility that exposure in the most sensitive population group (infants) exceeds the set TDI value, so a study of new technological strategies to reduce or prevent the formation of these contaminants is justified. However, it should be considered that the exposure time is very limited in this population, since the WHO recommends exclusive breastfeeding up to the first 6 months of life, when the inclusion of other foods begins. On the other hand, although other exposed population groups or groups that exceed the TDI values according to the estimates of exposure in the EFSA and JECFA reports, are children between 2 and 10 years, the data of real intake derived from ANIBES or ENALIA studies performed in Spain show a low intake of the food groups containing higher amounts of these contaminants [42,112,113].

In the EU, the maximum levels of various contaminants in foods (nitrates, mycotoxins, metals and 3-MCPDs, dioxin, PCBs, PAH, melamine, etc.) are governed by Regulation 1881/2006 [114]. This in turn is amended by Regulation (EU) 2018/290, which additionally provides maximum levels of GEs in oils and vegetable fats as well as follow-on milks and infant milks. Maximum 3-MCPD levels are only set for hydrolyzed vegetable protein and soy sauce [115]. Currently, the Commission’s working groups are discussing new values for 3-MCPDs and their esters in fats and vegetable oils, including oil palm, which will probably be approved during 2019. Table 7 shows the maximum levels of 3-MCPDs and GEs laid down by Regulation 2018/290.

### 8.3. Palm Oil and Risk of Cancer

Some social alarm regarding palm oil and cancer risk has been prompted by two different issues. On the one hand, the International Agency for Research on Cancer (IARC) has classified 3-MCPD as a possible carcinogen (group 2B) and glycidol and its esters as probable carcinogens (Group 2A), while 2-MCPD has not been evaluated by this WHO-dependent agency to date [116,117,118]. Qualification as a potential carcinogen by the WHO should not be associated with the existence of a real risk, because we have to distinguish between hazard and risk. The WHO says ‘danger’, but proper and transparent “case by case” risk assessment is needed to conclude that there is actually such a hazard. The health effects of these technologically-generated chemical contaminants are still the subject of discussion [100,118,119,120].

Then again, in 2017 Pascual et al., published a study showing that palmitic acid, or a high-fat diet, can facilitate certain metastasis mechanisms through expression of the CD36 (Cluster of differentiation 36) receptor [57]. Since then, attention has been focused on palmitic acid, and by association on palm oil. It is important to clarify that palmitic acid is not a toxic substance, as the authors have stated. Note that this is an in-vitro mechanistic study on expression of a receptor cell in tumor tissue with very high, not physiological, dose. This type of studies cannot be extrapolated to the amount of dietary FAs available for cells in-vivo.

Currently, there is no scientific evidence on the relationship between palm oil intake and the occurrence of cancer. According to the conclusions of the “Symposium of the Italian Foundation of Nutrition on palm oil and health” held in Milan from 3 May 2016, there is no experimental or epidemiological evidence to warrant associating the consumption of palm oil with a higher incidence of cancer mortality in human beings, and the indirect evidence suggests that palm oil has no positive or negative effects on the risk of cancer [3].

## 9. Palm Oil as an Example of the Complexity of Communication in Nutrition

Nutrition is a complex science, since it includes genetic, metabolic, biochemical and other aspects, as explained throughout this consensus document. In trying to convey this knowledge to the population, the media tend to simplify, omitting important details, which ultimately leads to disinformation or misinformation and even unfounded alarms. This problem is magnified nowadays by the ease of dissemination and access to information through social networks.

In fact, due to the controversy that has arisen regarding the safety of palm oil, the Committee on Nutrition of the Spanish Paediatrics Association (AEP) and the Spanish Society of Paediatric Gastroenterology, Hepatology, and Nutrition (SEGNP) issued a statement regarding the inclusion of palm oil and palmitic acid in infant formula, indicating that the restriction of fat intake, recommended as a general measure for improving the health of the general population, should not apply to infants and young children, and also that the incorporation of palmitate to infant formulas is necessary to ensure that the composition is as similar as possible to the composition of breast milk [121].

In order to analyze the perception of European consumers in relation to “no” or “free” labels on foods and beverages, the European Food Information Council (EUFIC) conducted a survey in 2017, where the three main aspects considered were the nutritional value of the product, environmental concerns and the safety of food for consumption. It was noted that consumers in countries such as the United Kingdom, France, or Sweden associated the term “palm oil” with environmental and ethical issues such as the destruction of the natural environment and the plight of orangutans; while in Poland it was associated with “healthier”. When analyzing consumer attitudes to labelling, it was noted that in France the preference was for products labelled “palm oil-free” as healthier, whereas in other countries there were no differences in this aspect of labelling. It is worth noting that of the countries surveyed, France is the one where the presence of products labelled “palm oil-free” is greatest.

Another aspect that can contribute to the confusion is when new knowledge arises in nutrition which questions or qualifies existing recommendations [122,123,124]. For instance, there is some recent meta-analysis that does not confirm an association between palm oil/SFA consumption and greater CVR or mortality in general [53,54,123,125,126]. Some authors like Mensink et al., have actually pointed out that SFAs also increase HDLc [127]. These new discoveries, which partly contradict previous results, disturb and puzzle the consumer. Therefore, scientific communication in a truthful but accessible language is fundamental.

## 10. Conclusions

### 10.1. Sustainability

1. In response to the environmental impact posed by the cultivation of oil palm, 15 years ago the RSPO was founded, which certifies the sustainability of palm oil production.

2. The “Amsterdam Declaration” urges the inclusion of sustainability throughout the food production chain. Several countries and organizations are signatories, among them the RSPO, which made a commitment to the implementation of 100% sustainable palm oil in the European food chain by 2020.

3. Thanks to the work done over the past years, palm oil is one of the agricultural products leading sustainable production, although concerns about sustainability are evident also in various crops globally. It is essential to educate the consumer on the importance of selecting sustainable products to contribute to maintenance of the environment and local economies in the producing countries.

### 10.2. Nutrition

1. The review of the current scientific evidence on the safety of palm oil indicates that its consumption in moderate amounts, and in the context of a varied, balanced and adequate diet, does not present health risks.

2. Several more-or-less current studies are contributing to a reassessment of strongly implanted dogmas about saturated fat and its relationship to cardiovascular health. If these recent studies are confirmed, the association between consumption of SFAs and CVR could be modified or at least qualified.

3. Currently there is not enough evidence to warrant modifying fat intake recommendations regarding 20%–35% of total calories, nor the criterion that SFA intake should not exceed 10% of the total energy intake, which means that this fat intake should be at most about 20 g/day within a 2000 kcal a day diet.

### 10.3. Industry

1. The food industry is among those most concerned to maintain a high level of product quality and satisfy consumer demands. Among these demands as they relate to palm oil, apart from nutritional aspects, there is increasing stress on food security, and to a lesser extent sustainability.

2. The fact is that the industry is already consuming sustainable palm oil, and the forecast is for a gradual increase in the use of RSPO certified palm oil, refined using milder processes to achieve a significant reduction in the presence of technologically-generated chemical contaminants.

3. The collaboration of universities, research and technological centers, and the media is essential to assure the dissemination of objective, unbiased information.

### 10.4. Safety

1. For the present there is no scientific evidence (experimental or epidemiological) to warrant associating palm oil consumption with a higher cancer incidence or mortality risk in humans.

2. Both the JECFA and the EFSA have established levels of TDI for toxic contaminants generated in the processing of vegetable oils (3-MCPDs and GEs) at 4 µg/kg bw/d and 2 µg/kg bw/d respectively. In light of these recommendations, the Commission has amended its regulation of contaminants regarding GEs and is working on 3-MCPDs.

## Figures and Tables

**Table 1:** Sustainable development goals (SDGs) (UN 2015–2030) [16].

**Goal 1**	**Goal 7**	**Goal 13**
No poverty	Affordable and clean energy	Climate action
**Goal 2**	**Goal 8**	**Goal 14**
Zero hunger	Decent work and economic growth	Life below water
**Goal 3**	**Goal 9**	**Goal 15**
Good health and well-being	Industry, innovation, and infrastructure	Life on land
**Goal 4**	**Goal 10**	**Goal 16**
Quality education	Reducing inequality	Peace, justice, and strong institutions
**Goal 5**	**Goal 11**	**Goal 17**
Gender equality	Sustainable cities and communities	Partnerships for the goals
**Goal 6**	**Goal 12**	
Clean water and sanitation	Responsible consumption and production	

**Table 2:** Roundtable for Sustainable Palm Oil (RSPO) principles [18].

1. Behave ethically and transparently
2. Operate legally and respect rights
3. Optimize productivity, efficiency, positive impacts and resilience
4. Respect community and human rights and deliver benefits
5. Support smallholder inclusion
6. Respect workers’ rights and conditions
7. Protect, conserve and enhance ecosystems and the environment

**Table 3:** Different criteria for fat intake recommendations for healthy adult population.

	WHO/FAO (2010) [37]	FESNAD (2015) [41]	AHA (2000) [40]
Total fat	20%–35%	20%–40%	30%
SFA	<10%	The recommendations are reducing the SFA-rich foods intake.	<10%
MUFA	Calculated by difference	12%–30% (27–67 g/day) 20%–25% (45–55 g/day) *	Calculated by difference
PUFA	6%–11%		<10%
PUFA W3 Alfa linolenic acid DHA + EPA		0.1%–1% (0.25–2.25 g/day) 0.5–1%	
PUFA W6	0.5%–2%	5%–10% (10–20 g/day)	
Trans-FAs	<1%		2%–3% Reduce the intake as much as possible
Cholesterol			<300 mg/day

* PREDIMED study data. Modified from Gesteiro et al., (2018) [39]. Fatty acids (FAs): saturated (SFA), monounsaturated (MUFA); and polyunsaturated (PUFA).

**Table 4:** Fatty acid composition (%) of the different palm oil and kernel oil fractions.

Fatty Acid	Palm Oil	Palm Stearin	Palm Olein	Palm Superolein	Palm Kernel Oil	Palm Kernel Stearin	Palm Kernel Olein
C6:0	ND	ND	ND	ND	ND-0.8	ND-0.2	ND-0.7
C8:0	ND	ND	ND	ND	2.4–6.2	1.3–3.0	2.9–6.3
C10:0	ND	ND	ND	ND	2.6–5.0	2.4–3.3	2.7–4.5
C12:0	ND-0.5	0.1–0.5	0.1–0.5	0.1–0.5	45.0–55.0	52.0–59.7	39.7–47.0
C14:0	0.5–2.0	1.0–2.0	0.5–1.5	0.5–1.5	14.0–18.0	20.0–25.0	11.5–15.5
C16:0	39.3–47.5	48.0–74.0	38.0–43.5	30.0–39.0	6.5–10.0	6.7–10.0	6.2–10.6
C16:1	ND-0.6	ND-0.2	ND-0.6	ND-0.5	ND-0.2	ND	ND-0.1
C17:0	ND-0.2	ND-0.2	ND-0.2	ND-0.1	ND	ND	ND
C17:1	ND	ND-0.1	ND-0.1	ND	ND	ND	ND
C18:0	3.5–6.0	3.9–6.0	3.5–5.0	2.8–4.5	1.0–3.0	1.0–3.0	1.7–3.0
C18:1	36.0–44.0	15.5–36.0	39.8–46.0	43.0–49.5	12.0–19.0	4.1–8.0	14.4–24.6
C18:2	9.0–12.0	3.0–10.0	10.0–13.5	10.5–15.0	1.0–3.5	0.5–1.5	2.4–4.3
C18:3	ND-0.5	ND-0.5	ND-0.6	0.2–1.0	ND-0.2	ND-0.1	ND-0.3
C20:0	ND-1.0	ND-1.0	ND-0.6	ND-0.4	ND-0.2	ND-0.5	ND-0.5
C20:1	ND-0.4	ND-0.4	ND-0.4	ND-0.2	ND-0.2	ND-0.1	ND-0.2
C20:2	ND	ND	ND	ND	ND	ND	ND
C22:0	ND-0.2	ND-0.2	ND-0.2	ND-0.2	ND-0.2	ND	ND
C22:1	ND	ND	ND	ND	ND	ND	ND
C22:2	ND	ND	ND	ND	ND	ND	ND
C24: 0	ND	ND	ND	ND	ND	ND	ND
C24:1	ND	ND	ND	ND	ND	ND	ND

Source: Codex Alimentarius (2015) [49].

**Table 5:** Reviews on the effect of palm oil on cardiovascular health.

Reference	Type of Study and Aims	Results and Conclusions
Bester et al. [78]	Review. To identify the benefits and risks of four edible oils (olive, sunflower, fish and palm oils) for cardiovascular health.	The studies reviewed here suggest that all four oils could be suitable for inclusion in a healthy diet. Beneficial effects have been identified for each one, although in some cases there remains some dispute.
		In the case of palm oil, the bulk of the studies have addressed its effects on the serum lipid profile, concluding that the lipid profile is unaffected, and can even reduce atherosclerosis and prevent cardiac ischaemia. The few studies addressing the effect of palm oil on arrhythmogenesis are inconclusive, although it can apparently have a slight anti-arrhythmogenetic effect.
		The studies performed with extracts of the tocotrienol-rich fraction of palm oil suggest that it may offer protection against myocardial ischaemia-reperfusion injury. However, these effects require further study with dietary supplementation models.
		Studies with red palm oil (RPO), which contains the same components as refined oil but also contains more micronutrients, confirm the above-described effects of palm oil, enhanced by the presence of more micronutrients. It has also been shown that dietary supplementation with RPO offers protection against myocardial ischaemia-reperfusion injury. Again, however, further studies with RPO are needed to fully confirm these effects on risk factors of ischaemia and cardiac arrhythmias.
		In conclusion, palm oil has shown only a mild effect on the lipid profile, and so there is no saying whether its consumption would have any beneficial effect in persons at cardiovascular risk (CVR). Nonetheless, it could be useful for some people to consume this oil, since it is one of the few oils to have demonstrated protective effect against myocardial ischaemia-reperfusion injury.
Teng et al. [79]	Single-blind randomized crossover trial (n = 10 healthy males) comparing the effects of high intake of fat (50 g) rich in palmitic acid of both plant (palm olein) and animal (pork lard) origin versus consumption of a fat rich in oleic acid (virgin olive oil) on lipaemia, plasma glucose, insulin and adipocytokines.	The serum triglyceride (TG) concentration was significantly lower after consumption of pork lard than after consumption of olive oil or palm olein (meal effect *p* = 0.003; time effect *p* < 0.001). There was also a greater reduction in plasma levels of non-esterified free fatty acids (FFA) in the group that consumed pork lard than in the group that consumed olive oil (*p* < 0.05). The differences in the physical and structural characteristics of pork lard TGs may account to some extent for the fact that the increase of postprandial lipaemia was smaller than in the case of palm olein. The effects of palm olein and olive oil on postprandial lipaemia were similar.
		Levels of plasma glucose, insulin and adipocytokines [interleukin-6 (IL-6), tumoral necrosis factor-α (TNF-α), interleukine-1β (IL-1β) and leptin] were unaffected by the type of fat consumed. In the case of plasma IL-1β, there was such a change over time after taking 3 meals with a high-fat diet (*p* = 0.036). These results could suggest that it is a high fat intake, rather than the type of fatty acid (FA), that affects the response of pro-inflammatory markers.
Fattore and Fanelli [80]	Review of the scientific evidence on the relationship between palm oil and adverse health effects.	The main reason why consumption of palm oil is associated with adverse health effects is that it contains relatively high concentrations of saturated fatty acids (SFAs), particularly palmitic acid, which in turn have been associated with increased risk of coronary heart disease (CHD) and some types of tumor. However, recent research on the subject has reconsidered the negative role of dietary SFAs as a CVR factor and has shown that not only the type of fat but also the structure of the TGs plays an important part in cholesterolaemia.
		Some studies have concluded that modification of the type of fat, or modification combined with reduction (but not only reduction) appear to reduce the incidence of cardiovascular events in high-risk subjects. Moreover, most studies posit a possible protective effect from partial replacement of SFAs by polyunsaturated (PUFAs) as opposed to the adverse effect of SFAs *per se*. This is indicative of the importance of continuing research to improve the balance between SFAs and PUFAs in foods.
		The process of interesterification, which causes a reordering of palmitic acid and does not occur naturally in native oil, could be associated with potentially adverse health effects and hence is to be discouraged.
		As for its role in the development of cancer, the studies are few and the evidence unconvincing.
		Despite these uncertainties, this review does not demonstrate a negative role of palmitic acid in health, and much less native palm oil, which is a complex alimentary matrix in which palmitic acid is only one of the components. Palm oil also contains other FAs, chiefly oleic acid, along with antioxidant compounds, which may have compensatory effects.
Fattore et al. [52]	Systematic review and meta-analysis of dietary intervention assays, to evaluate the effect on lipid markers for CHD and cardiovascular disease (CVD) of replacing palm oil with other primary dietary fats. The review includes a total of 51 intervention studies ranging in duration from 2 to 16 weeks, in which palm oil-rich diets were compared with diets rich in other fats, and in which at least one or more of the following CHD or CVD biomarkers were determined: total cholesterol (TC), cholesterol transported by low density lipoproteins (LDLc), cholesterol transported by high density lipoproteins (HDLc), TC/HDLc ratio, TG, apolipoprotein (Apo) A-I and Apo B.	Levels of TC, LDLc, Apo B, HDLc and Apo A-I were significantly higher in palm oil-rich diets than in diets rich in stearic acid, monounsaturated fatty acids (MUFAS) and PUFAs whereas most of these biomarkers were significantly lower than in diets rich in myristic and lauric acids.
		Comparison of the effect of palm oil-rich diets with diets rich in trans-FAs showed that HDLc and Apo-A-I concentrations were significantly higher, while Apo B, and TG concentrations and TC/HDLc ratio were lower.
		This meta-analysis indicates that replacement of palm oil by other fats (SFAs, MUFAs, PUFAs) produces both favorable and unfavorable changes in biomarkers for CVD and CHD. However, when palm oil was replaced by trans-FAs, only favorable changes were found.
		The results do not support an association between palm oil replacement and reduction of mortality from CVD. They rather suggest a need for fresh comparative research and also call for caution in the development of policies for the general population that promote the use of some specific fats over others. More solid evidence is required as to the effects of palm oil on health and on the socio-economic consequences before embarking on such policies.
Voon et al. [81]	Randomized crossover trial of 5 weeks with a total of 45 volunteers. To assess the effects of a (typical Malaysian) diet containing a high proportion of protein and supplemented with virgin olive oil, palm olein or coconut oil on arteriosclerosis markers (cell adhesion molecules, lipid inflammatory mediators and thrombogenicity indices) in healthy adults.	SFA-enriched diets (palm olein and coconut oil), and also a diet with virgin olive oil, containing high levels of oleic acid, have similar effects on arteriosclerosis markers, such as cell adhesion molecules and prostaglandin E2 (PGE2) and thromboxane B2 (TXB2)/Prostaglandin F1α (PGF1α) thrombogenicity indices.
		Only the diet supplemented with virgin olive oil exhibited a lower level of the leukotriene B4 (LTB4) pro-inflammatory marker than the other two diets assayed.
		The results indicate that it could be premature labelling palm olein and coconut oil as “bad oils” because of their contribution to increased CVR based solely on their effects on the serum lipid profile.
Odia et al. [82]	Review of experimental studies in animals and humans on the association between palm oil and its constituents and serum lipid profile and CVD.	Many scientific studies, both in animals and human beings, clearly show that consumption of palm oil does not cause a rise in serum TC levels and that it is not atherogenic. Apart from palmitic acid, palm oil is composed of oleic and linoleic acids and also contains vitamins A and E, which are powerful antioxidants. It has been demonstrated scientifically that palm oil protects the heart and blood vessels from plaque and ischaemic lesions.
		Consumed as part of a healthy, balanced diet, it does not cause increased CVR. Replacing this oil with others rich in MUFAs and PUFAs would not produce any additional benefit.
		This review concludes that more longitudinal studies are required to assess the impact on CVR of diets containing palm oil in comparison with diets containing other oils accepted as “beneficial for cardiovascular health”, such as olive oil, analyzing lipid parameters as risk markers.
Imoisi et al. [83]	Review on palm oil: composition and health implications.	Prospective epidemiological studies have shown that the level of plasma TGs, especially after eating, is a major factor in the pathogenesis of CHD. Palm oil is considered as to raise TC and to increase the CVR because of its SFA content (44% palmitic acid and 5% stearic acid). However, there is a growing body of scientific evidence indicating that the effect of palm oil on TC is relatively neutral compared to other fats and oils. Palm oil raises TC only when an excess of dietary cholesterol is present in the diet.
		Palm oil stimulates the synthesis of HDLc and the elimination of LDLc.
		Palm oil is rich in vitamin E (particularly tocotrienols), which can reduce serum TC concentrations and has powerful antioxidant effects. These benefits occur especially from non-oxidized palm oil. Oxidized palm oil can have an adverse effect on lipid profile, FFAs and phospholipids.
		In conclusion, consumption of palm oil as a fat source in the diet does not pose any additional risk of CHD when consumed in realistic quantities as part of a healthy diet. Consumption of oxidized palm oil should be avoided owing to its adverse effects on lipid profiles.
Mancini et al. [84]	Review on the functional role of palm oil and palmitic acid in the development of obesity, type 2 diabetes mellitus, CVD, and cancer. It also discusses the atherogenic potential of palmitic acid and its stereospecific position in TGs.	As to the possible effects of palm oil-rich diets on CVR in humans, the studies reviewed here report conflicting results. The main criticisms of these may be summarized as follows: (a) considerable qualitative and quantitative heterogeneity of FA concentrations in the diets; (b) differences in selection criteria used to form the trial and control groups; (c) wide age range addressed in the studies; and (d) little attention is paid to other dietary components which could confound the direct effects of FAs on blood lipid markers.
		To date no clear evidence has been found to demonstrate beyond a doubt the association of consumption of palm oil with increased CVR, particularly in normo-cholesterolaemic subjects, assuming the recommended intake of PUFAs.
		Moreover, the percentage of palmitic acid at position sn-2 on the TG is smaller in palm oil than in animal fats, which supports the hypothesis that palm oil has little atherogenic potential in the context of a balanced diet, and that the alleged adverse effects may be due to a dose-response ratio.
		This review posit the need to carry out more rigorous research to define the advantages and disadvantages of palm oil consumption as regards CVR.
Sun et al. [60].	Systematic review on the effect of palm oil consumption on blood lipids compared with other cooking oils, based on data from clinical trials (minimum duration 2 weeks, comparing the effects of palm oil consumption with that of other oils such as; low-SFA vegetable oils, partially hydrogenated vegetable oils containing trans-FAs and animal fats).	Palm oil significantly increased LDLc by 0.24 mmol/L (95% CI: 0.13, 0.35 mmol/L; I(2) = 83.2%) compared with other low-SFA vegetable oils. This effect was observed in randomized but not in non-randomized trials. Moreover, among randomized trials, only modest differences in study results were observed regarding the effects of palm oil versus LDLc. SFAs from palm oil appear to have the same effects on LDLc as animal fat.
		Palm oil increased HDLc by 0.02 mmol/L compared with the low-SFA vegetable oils, and by 0.09 mmol/L compared with oils containing trans-FAs.
		In conclusion, the consumption of palm oil results in a higher level of LDLc than that of low-SFA vegetable oils, and in a higher level of HDLc if compared with the effect of oils containing trans-SFAs. This would tend to support the recommendation to reduce the use of palm oil and replace it with low-SFA and trans-FA vegetable oils.
		In the case of foods that are consumed in small quantities which are made using trans-FAs for their sensory characteristics, palm oil can be an alternative thanks to its better effects on HDLc.
Marangoni et al. [3]	Summary of the symposium‘s conclusions on the use of palm oil in the food industry, produced by experts from several Italian medical and nutritional scientific societies and collected by the nutrition foundation of Italy. Toxicological and environmental issues were not considered.	The main conclusions of the Symposium may be summarized thus:
		(a) There is no evidence on the specific health effects of palm oil consumption as compared with other SFA-rich fats.
		(b) The stereospecific distribution of SFAs in the TG molecules of palm oil limits their rate of absorption and their metabolic effects.
		(c) International guidelines indicate that SFA intake should be less than 10% of total energy, within a balanced diet. Within these limits, there is no reason to expect palm oil consumption to have any effect on human health (and especially on CVD or cancer risk).
		Palm oil intake does not appear to be a public health priority in Italy, in light of its overall effects and available consumption data.

Apo, apolipoprotein; CHD, coronary heart disease; CVD, cardiovascular disease; CVR, cardiovascular risk; FAs, fatty acids; FFAs, free fatty acids; HDLc, cholesterol transported by high density lipoproteins; IL, interleukin; LDLc, cholesterol transported by low density lipoproteins; MUFAs, monounsaturated fatty acids; PUFAs, polyunsaturated fatty acids; RPO, red palm oil; SFAs, saturated fatty acids; TC, total cholesterol; TG, triglyceride.

**Table 6:** 3-MCPD and glycidil ester (GE) levels in different oils and fats.

	Mean Content (mg/kg) (2009)	Maximum Content (mg/kg) (2009)	Mean Content (ppm) (2011)	Maximum Content (ppm) (2011)	Mean Content (mg/kg) (2019)	Maximum Content (mg/kg) (2019)
Canola oil	0.3	1.5	1	1	1.03 (*)	N
Soya oil	N	N	0.5	0.6	N	N
Sunflower oil	1	5.7	2	4	1.82	2.05
Corn oil	2.8	7	7	9	0.9	1.55
Coconut oil	N	N	7	7.5	Nd	Nd
Palm oil	4.5	13	6	14	6.22 (*)	N
Sesame oil	N	N	N	N	1.6	1.63
Safflower oil	N	N	N	N	2.78 (*)	N
Extra virgin olive oil	N	N	N	N	Nd	Nd
Refined olive oil 1°	N	N	N	N	0.78	1.11
Refined olive oil 0.4°	N	N	N	N	0.95	1.22
Fried extra virgin olive oil	N	N	N	N	0.93	1.72

Prepared by authors, based on Mathäus et al., 2011 [104]; Willits, 2013 [105]; and Custodio-Mendoza et al., 2019 [106]; N: non- determined; Nd: Non detectable; (*) Data from a single oil sample.

**Table 7:** Maximum levels of 3-MCPD and GE (Regulation 2018/290) [115].

Foodstuffs		Maximum Level (μg/kg)
**4.1**	**3-Monochloropropanediol (3-MCPD)**	
4.1.1	Hydrolysed vegetable protein	20
4.1.2	Soy sauce	20
**4.2**	**Glycidyl Fatty Acid Esters Expressed as Glycidol**	
4.2.1	Vegetable oils and fats placed on the market for the final consumer or for use as an ingredient in food with the exception of the foods referred to in 4.2.2	1000
4.2.2	Vegetable oils and fats destined for the production of baby food and processed cereal-based food for infant and young children	500
4.2.3	Infant formula, follow-on formula and foods for special medical purposes intended for infants and young children (powder)	75 until 30 June 2019. 50 as from 1 July 2019
4.2.4	Infant formula, follow-on formula and foods for special medical purposes intended for infants and young children (liquid)	10.0 until 30 June 2019. 6.0 as from 1 July 2019
